# Impaired neuropsychological functioning in patients with hypopituitarism

**DOI:** 10.1002/edm2.165

**Published:** 2020-06-29

**Authors:** Tessa N. A. Slagboom, Jan Berend Deijen, Christa C. Van Bunderen, Hans A. Knoop, Madeleine L. Drent

**Affiliations:** ^1^ Amsterdam UMC, Vrije Universiteit Amsterdam, Department of Internal Medicine, Section of Endocrinology, Amsterdam Neuroscience Amsterdam The Netherlands; ^2^ Hersencentrum Mental Health Institute Amsterdam The Netherlands; ^3^ Section of Clinical Neuropsychology Department of Clinical, Neuro‐ & Developmental Psychology Faculty of Behavioral and Movement Sciences Vrije Universiteit Amsterdam The Netherlands; ^4^ Amsterdam UMC, University of Amsterdam, Department of Medical Psychology Amsterdam The Netherlands

**Keywords:** cognition, executive function, hypopituitarism, information processing, memory, neuropsychology, pituitary

## Abstract

**Background:**

Treatment of pituitary pathology mostly does not result in complete recovery of impairment in cognitive functioning. The primary aim of the current study was to assess cognitive impairment in patients with stable replacement therapy for hypopituitarism during the last 6 months prior to inclusion. It was expected that patients showed subjective and objective subnormal scores on neuropsychological functioning.

**Methods:**

Forty‐two patients (40% men, 49 ± 15 years) treated for hypopituitarism conducted a neuropsychological test battery, including the Cognitive Failures Questionnaire (CFQ), 15‐Word test (15‐WT), Cambridge Neuropsychological Test Automated Battery (CANTAB) Motor Screening Task (MOT), Spatial Working Memory (SWM) and Affective Go/No‐go (AGN). Results were compared to reference values of healthy norm groups.

**Results:**

Male and female participants scored significantly worse on the CFQ (*P* < .01, *d* = 0.91‐4.09) and AGN mean correct latency (*P* < .01, *d* = 1.66 and 1.29, respectively). Female participants scored significantly worse on 15‐WT direct recall (*P* = .01, *d* = 0.66), 15‐WT delayed recall (*P* = .01, *d* = 0.79), SWM total errors (*P* = .05, *d* = 0.41), SWM strategy (*P* = .04, *d* = 0.43), AGN errors of commission (*P* = .02, *d* = 0.56) and omission (*P* = .04, *d* = 0.41).

**Conclusion:**

This study shows that subjective cognitive functioning is worse in patients treated for hypopituitarism compared to reference data. Also, female participants treated for hypopituitarism score worse on objective aspects of memory and executive functioning compared to reference data. Besides worse focus attention, this objective cognitive impairment was not found in male participants. It is recommended to conduct additional research, which focuses on the design and evaluation of a cognitive remediation therapy, aimed at compensation of impairments in different aspects of memory and executive functioning.

## Graphical abstract

Treatment of pituitary pathology mostly does not result in complete recovery of impairment in cognitive functioning. This study shows that subjective cognitive functioning is worse in patients treated for hypopituitarism compared to reference data. Also, female participants treated for hypopituitarism score worse on objective aspects of memory and executive functioning compared to reference data.

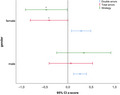

## 1 INTRODUCTION

Hypopituitarism is a rare condition characterized by a deficit of one or more of the pituitary hormones. Clinical manifestations of hypopituitarism vary widely since it represents a diverse group of conditions, including differences in aetiology, pathology and hormonal deficiencies. Treatment of hypopituitarism consists of hormone substitution therapy. Unfortunately, current substitution methods do not completely mimicry the endocrine homeostasis and they are titrated based on plasma variables and not on action at tissue level.^1^ Therefore, subtle derangements in hormonal status may appear in patients treated with substitution therapy, which in turn can lead to vague complaints and thereby a decreased quality of life.^1^ Ehrnborg et al^2^ found that patients with hypopituitarism, compared to the general population, had more health‐related costs, took more sick leave days and were more likely to claim a disability pension. Studies also found a decreased mood state in patients treated for hypopituitarism.^3^, ^4^ Altogether, patients with hypopituitarism still experience problems and effects of their illness, even after treatment of their hormonal disturbances.

Most of the pituitary (stimulating) hormones and their products can cross the blood‐brain barrier and connect to specific binding sites throughout the central nervous system. This finding suggests that hormones act on the brain and that hormone deficiencies can have an effect on brain functioning and structure. Studies have indeed shown that hormonal deficiencies negatively affect neuropsychological functioning, such as memory and executive functioning.^5^, ^6^, ^7^

The finding of a compromised neuropsychological functioning in patients with pituitary disease can still be found after adequate replacement therapy. Scientific research showed impairments in memory,^8^, ^9^ executive functions,^8^, ^9^, ^10^ attention^8^, ^11^ and visuospatial processing^8^ in patients following successful treatment of Cushing’s disease (CD). Structural brain abnormalities in these patients may attribute to their impaired neuropsychological functioning.^12^, ^13^, ^14^ Hypopituitarism in patients with effective treatment of CD was associated with mildly impaired executive functioning.^9^ For patients with successful treatment of acromegaly, a decrease in memory is also found, which is associated with a decreased activity in the left medial temporal cortex.^15^ The same study of Martín‐Rodríguez et al^15^ showed a trend of decrease in performance on a visual memory task and less activity in the left medial temporal cortex for patients with hypopituitarism after treatment of acromegaly. Besides a decrease in memory, patients in remission from acromegaly also show a decrease in executive functioning.^16^ It could be stated that this neuropsychological deterioration is the result of previous hormonal overproduction in CD and acromegaly, but the same neuropsychological deterioration is found in patients treated for nonfunctioning pituitary macroadenoma (NFPA),^17^ a condition without hormonal overproduction. Another study on patients treated for NFPA found that these patients show a high self‐reported prevalence and severity of cognitive dysfunction.^18^ Hence, it seems that cure of pituitary disorder does not result in complete recovery of neuropsychological function, which can be due to structural brain changes by previously hormonal over‐ and/or underproduction and/or shortcomings in treatment.

Some of the long‐term effects of hypopituitarism on cognitive functioning are known for different aetiologies of hypopituitarism, as described by the studies mentioned above. Most research in this field has been directed on a restricted range of cognitive domains within specific subgroups of hypopituitarism. However, during clinical practice, endocrinologists face patients with all different kinds of aetiologies of hypopituitarism. The aim of the current study, therefore, was to assess a spectrum of cognitive functions in a heterogeneous group of patients with hypopituitarism with subjective and objective measures, at least six months after adequate replacement therapy. These insights are of importance because they can provide essential information for clinical practice about the course of hypopituitarism and the expected long‐term problems on cognitive functioning, which patients might encounter in general. Also, no other studies have been performed on both subjective and objective cognitive functions within this patient group. This study might provide more insight in what specific subdomains patients treated for hypopituitarism could underperform. It was expected that these patients show impaired scores on neuropsychological tasks.

## 2 MATERIALS AND METHODS

### 2.1 Participants

All participants (N = 42) were patients with hypopituitarism from the Section of Endocrinology, Department of internal medicine, Amsterdam UMC, Vrije Universiteit Amsterdam, at least 6 months after adequate replacement therapy. Hypopituitarism was defined as a deficit of one or more of the pituitary hormones. Pituitary function was assessed by clinical manifestations and biochemical testing at least twice a year during outpatient clinic visits, and deficits were treated adequately. Laboratory results of hormonal levels of the participants are given in Table 1. Pituitary deficits were defined according to international guidelines at the time of diagnosis.^19^ Central growth hormone and adrenal deficiency were diagnosed by dynamic testing: either by an insulin tolerance test (GH and cortisol), GHRH‐arginine test (GH), CRH test (cortisol) or Synacthen test (cortisol). Cut‐off scores defined by the Endocrine Society Clinical Practice Guideline at the time of diagnosis were used.^19^ Inclusion criteria for this study were the diagnosis of hypopituitarism, age between 18 and 70 years and entry in the study at least 6 months after stabilizing of the hormonal disturbances. Replacement therapy was classified as adequate if biochemical testing showed that levels of pituitary hormones and/or their end‐products were above cut‐off scores defined by the Endocrine Society Clinical Practice Guideline.^19^ Patients who were mentally retarded, suffering from dementia or severe (cognitive) illness or chronic users of medication that influences consciousness were not included. Table 2 shows participant demographics and clinical characteristics. None of the participants received a compensation for participating in the study. Testing started in January 2018 and ended in April 2018. The Medical Ethical Committee of the Amsterdam University Medical Center, Vrije Universiteit Amsterdam, approved the protocol.

**Table 1:** Laboratory results of hormone levels of participants during last check‐up

	N	*M* (SD)	Reference range	Unit	Lab method
fT4	42	17.66 (2.93)	12‐22	pmol/L	Immunoassay Cobas (Roche Diagnostics)
IGF‐1 SD	42	0.19 (1.16)	−2.0 to 2.0		Chemiluminescence immunoassay Liaison (Diasorin S.p.A)
Testosterone	17	19.38 (9.00)	>8.0	nmol/L	2nd generation testosterone assay Architect (Abbott Diagnostics)

Abbreviations: fT4, free thyroxine; IGF‐1, insulin‐like growth factor‐1.

**Table 2:** Participant demographics and clinical characteristics

	n (%)
Gender (males/females)	17/25
Age (y), mean ± SD	49 ± 15
Duration of disease^a^ (y), mean ± SD	22 ± 12
Education level^b^ (1/2/3/4/5/6/7)	0/0/1/4/19/9/9
Previous treatment (surgery/radiotherapy/chemotherapy)	35/15/2
Cause of hypopituitarism	
Congenital^c^	5 (12%)
Acquired	
Nonfunctioning pituitary adenoma	10 (24%)
Craniopharyngioma	6 (14%)
Acromegaly	5 (12%)
Cushing’s disease	5 (12%)
Prolactinoma	4 (10%)
Other	7 (17%)
Pituitary axis deficiency/hormone substitution	
Pituitary deficiencies: 1/2/3/4/5	4/8/9/10/11
No. hormone replacements: 1/2/3/4/5	4/10/14/9/5
ACTH deficiency/cortisol substitution	32(76%)/32(76%)
TSH deficiency/T4 substitution	34(81%)/34(81%)
FSH/LH deficiency/estradiol/testosterone substitution	30(71%)/19(45%)
GH deficiency/GH substitution	32(76%)/28(67%)
ADH deficiency/ADH substitution	14(33%)/14(33%)
Comorbidity	
No/yes	16/26
Hypertension	19 (45%)
Type 2 diabetes mellitus	4 (10%)
Epilepsy	4 (10%)
Dyslipidaemia	3 (7%)
Osteoporosis	3 (7%)
Asthma	2 (5%)
Other	10 (24%)
Use of other medications	
No/yes	12/30
Antihypertensive	14 (33%)
Antidepressant	1 (2%)
Antiepileptic	5 (12%)
Diabetic	4 (10%)
Statin	9 (21%)

Abbreviations: ACTH, adrenocorticotropic hormone; ADH, antidiuretic hormone; FSH, follicle stimulating hormone; GH, growth hormone; IGF‐1, insulin‐like growth factor‐1; LH, luteinizing hormone; T4, thyroxine; TSH, thyroid‐stimulating hormone.

^a^ Duration of disease: according to the date on which hypopituitarism was diagnosed.

^b^ According to Verhage^22^: 1 (elementary school not finished) to 7 (university level).

^c^ Other: medulloblastoma (two participants), meningioma, optic glioma, Tolosa‐Hunt syndrome, infectious: meningitis and tuberculosis.

### 2.2 Materials

#### 2.2.1 Subjective cognitive functioning

Complaints with respect to cognitive functioning were assessed with the Cognitive Failures Questionnaire (CFQ). The CFQ^20^ is a questionnaire consisting of 25 items about minor, everyday mistakes. Patients were asked to rate the frequency of different mistakes (eg, bumping into other people, forgetting names of other people) on a five‐point scale (1‐5), from “never” to “very often”. The CFQ has four subscales: absent‐mindedness, absent‐mindedness during social situations, forgetting of names and words and orientation. A higher score represented more mistakes, hence more complaints. Additionally, four questions were asked about the everyday mistakes: has there been an increase during the last 5 years, to what extent lead the mistakes to limitations in daily life, to what extent do they make you worry and to what extent do they annoy you. All questions were answered on a five‐point Likert scale with answers from “not at all” to “very strongly”. The CFQ has been validated in 1358 healthy subjects between 24 and 86 years. The questionnaire can be applied in clinical and nonclinical studies on cognitive functioning.^21^ Reference values from an age‐related Dutch population were derived from a study of Ponds et al^21^ The reference population consists of the young middle‐aged subgroup of previous mentioned study, with a total of 365 healthy Dutch participants (49% men). Mean age was 45.5 years (SD = 4.1), and average educational level was 3.9 (SD = 1.8) according to the system of Verhage.^22^

#### 2.2.2 Short‐ and long‐term memory

The 15‐Word test (15‐WT)^23^ was used to measure different aspects of verbal memory: short‐term memory (direct recall) and long‐term memory (delayed recall). In the direct recall condition, the investigator read 15 words out loud, after which the patient had to remember and repeat as much words as possible. This was done five times, and the score on the direct recall was the sum of the correctly remembered words for the five repeats (max score of 75). After 20 minutes, the delayed recall was administered, in which the patient was asked to name all the words on the list they remembered (max score of 15). For both conditions, a higher score is indicative for better memory functioning. Age‐, gender‐ and education‐ specific reference values were derived from Schmand et al^24^ The reference population consists of 847 healthy Dutch participants (55% women) with ages ranging from 14 to 87 years (*M* = 41.4, SD = 21.5). Average educational level was 5.0 (SD = 1.2) according to the system of Verhage.^22^ The 15‐WT is based on Rey’s Auditory Verbal Learning Test.^25^ It is one of the most frequently used memory tests in the Netherlands and has been validated by COTAN (www.cotandocumentatie.nl).

#### 2.2.3 Motor Screening Task

The first administered CANTAB^®^ test was the Motor Screening Task (MOT), which was used to introduce the software to the participant and to screen for sensorimotor deficits or lack of comprehension. During this task, participants touched crosses on different locations on the touchscreen as quickly and accurate as possible. Test administration time was 2 minutes. Outcome measures were speed of response and accuracy of pointing and higher scores mean more sensorimotor deficits or lack of comprehension. Age‐ and gender‐related reference values were derived from normative data provided by CANTAB.

#### 2.2.4 Spatial working memory and executive function

During the CANTAB^®^ Spatial Working Memory Task (SWM), participants were asked to fill up an empty column on a touchscreen with blue “tokens”, which they found under a series of coloured boxes. The more boxes on the screen, the more boxes the participants had to search through and the harder the test was. Next to working memory (eg, retention and manipulation of visuospatial information), this task also demanded executive functioning (eg, strategy). Administration time was estimated at 4 minutes. Outcome measures were double errors, total errors and strategy. A high score on double or total errors meant poorer working memory and executive functioning, and high scores on strategy represented worse executive functioning. Age‐ and gender‐related reference values were derived from normative data provided by CANTAB.

#### 2.2.5 Information processing biases and set‐shifting

The Affective Go/No‐go (AGN) CANTAB^®^ test^26^ took about 10 minutes and assessed information processing biases for positive and negative stimuli and set‐shifting. The test consists of 8 blocks, each of which included a series of 18 words from two different categories: positive (eg, joyful) or negative (eg, hopeless). The participant was given a target category and was asked to touch the press pad as quickly as possible, but only when a presented word matches this category. During a block, half of the words matched the category (targets) and the other halves were distractors. When pressed to a distractor stimulus, a tone sounded. Also, a distinction was made in blocks with or without set‐shifting: nonshift blocks (continue to respond to the same target category as the previous block, eg, the target category remains positive) and shift blocks (respond to the other category than the previous block, eg, the target category switches from positive to negative). First, two practice blocks in one of the two categories (ie, positive or negative) were computed. Patients were randomly assigned to a positive or negative practice block. Next testing started in which four nonshift blocks and four shift blocks were assessed. Outcome measures were latency (milliseconds) and number of errors of commission (incorrect responding to distracter stimuli) and omission (failure to respond to target stimuli) in total, positive, negative, shift and nonshift blocks. This test assesses inhibitory control on three levels of cognitive processing: one’s ability to focus attention and to inhibit behavioural responses (mean latency, errors of commission and omission), one’s ability to inhibit and reverse stimulus‐reward associations (difference between shift and nonshift blocks) and the existence of mood‐congruent attentional bias (difference between positive and negative blocks).^26^ Higher scores indicated more information processing biases. Reference values were derived from studies of Knight et al^27^ The CANTAB computerized test battery has been exclusively used in clinical practice as well as scientific studies in a wide range of disorders and healthy controls. It is mentioned in over 2000 scientific publications, and its ability to adequately discriminate between healthy adults and various (neuro)psychiatric populations has been confirmed.^28^, ^29^

#### 2.2.6 Procedure

Patients were consecutive and invited by mail by their endocrinologist. They received a phone call a couple of weeks later, in which an appointment was made. Before testing started, patients signed the informed consent. Patients performed the test battery in a quiet room. Testing found place during morning or afternoon. First, the patients were interviewed on their subjective complaints, with emphasis on complaints that had arisen or aggravated since pituitary pathology. Next, the 15‐Word test direct recall condition was administered followed by the CANTAB tests. After completion of the CANTAB tests, the 15‐Word delayed recall condition was administered. Finally, the CFQ was administered. The estimated time for completing the testing was one hour, and administration time of the cognitive tests was about 20 minutes. An investigator was present at all time.

#### 2.2.7 Data analysis

Descriptive statistics were used to describe the basic features of the data and to provide summaries of the sample and the measures. Primary study parameters of cognition were compared with reference data. In addition, statistical comparisons of demographic variables between participants and nonparticipants were done by *t* tests and chi‐squared tests. Comparisons against reference values of variables were performed by one‐sample *t* tests. Effect size was calculated as Cohen’s *d* (mean difference/SD). To ascertain that our sample provided enough power to detect the hypothesized effects, we conducted a post hoc power analysis for the t tests using the program G* power 3.1.9.4.^30^ After applying an effect size *d* = 0.5, the obtained power was 0.94.

For all statistical analyses, SPSS Statistics version 22 was used.

## 3 RESULTS

Out of the 101 patients who were invited to participate in the study, 42 patients (42%) participated. Amongst mentioned reasons for refusing participation were long journey distance, too stressful and no interest. Participants did not differ from nonparticipants on age (*t*(99) = 1.82, *P* = .07), gender (*χ* ^2^(1) = 0.48, *P* = .49), illness duration (*t*(99) = 1.36, *P* = .18) and number of pituitary deficiencies (*χ* ^2^(4) = 6.30, *P* = .18). They did differ on the origin of their hypopituitarism: of the nonparticipants, 20 (34%) had an congenital form of hypopituitarism versus 5 (12%) in the participants (*χ* ^2^(1) = 6.37, *P* = .01). Also, participants had significantly more often a history of surgery than nonparticipants (*χ* ^2^(1) = 15.82, *P* < .00), but not of radiotherapy (*χ* ^2^(1) = 2.29, *P* = .13) or chemotherapy (*χ* ^2^(1) = 0.01, *P* = .94). There were missing data for one participant on the AGN so this participant was excluded for results on the AGN.

### 3.1 Subjective cognitive functioning

Subjective cognitive functioning data are shown in Table 3. Both men and women scored significantly worse on the total CFQ and all CFQ subscales compared to reference means with large to very large effect sizes (*d* = 0.91‐4.09). On the extra questions, women had significantly higher scores, meaning they experienced a higher increase in, more hindrance of, worries about and were more often annoyed by their cognitive dysfunctions. Men did not score significantly different on the extra questions.

**Table 3:** Subjective cognitive functioning and comparisons to reference data

Cognitive failures questionnaire	Hypopituitarism		Reference Mean	*t*	*P*	*d*
	Range	Mean (SD)				
Total						
*♂*	40‐73	59.06 (9.82)	32.8	11.02	**<.01**	2.67
*♀*	28‐65	53.92 (12.58)		12.58	**<.01**	1.68
Subscales						
Absent‐mindedness						
*♂*	17‐32	25.59 (4.40)	7.8	16.66	**<.01**	4.04
*♀*	14‐29	23.28 (4.08)		18.98	**<.01**	3.79
Absent‐mindedness during social interactions						
*♂*	7‐19	13.29 (3.16)	6.0	9.52	**<.01**	2.30
*♀*	6‐17	12.84 (2.87)		11.93	**<.01**	2.38
Names and words						
*♂*	3‐13	8.12 (2.60)	5.6	4.00	**<.01**	0.97
*♀*	4‐11	7.16 (1.72)		4.52	**<.01**	0.91
Orientation						
*♂*	7‐15	12.06 (2.19)	3.1	16.84	**<.01**	4.09
*♀*	4‐14	10.64 (3.03)		12.46	**<.01**	2.49
Extra questions						
Increase						
*♂*	0‐4	1.94 (1.03)	1.7	0.97	0.35	0.23
*♀*	0‐4	2.56 (1.04)		4.12	**<.01**	0.82
Hindrance						
*♂*	0‐4	2.35 (0.98)	2.0	1.46	0.16	0.36
*♀*	0‐4	3.12 (1.05)		5.32	**<.01**	1.07
Worry						
*♂*	0‐4	2.18 (0.88)	1.9	1.29	0.22	0.32
*♀*	0‐4	2.68 (0.85)		4.58	**<.01**	0.92
Annoy						
*♂*	0‐4	2.24 (0.97)	1.9	1.43	0.17	0.35
*♀*	0‐4	2.92 (1.00)		5.12	**<.01**	1.02

Bold values represent *p* < .05.

### 3.2 Short‐ and long‐term memory

More than 50% of the female participants had a score below the 30th percentile on both memory subscales, and about 30% had a score below the 10th percentile. Scores of males were less often in these low percentiles, as shown in Table 4. Women scored significantly lower on the 15‐WT direct recall and 15‐WT delayed recall when compared to their reference sample, with medium effect sizes (*d* = 0.66 and 0.79, respectively). No significant differences were found for men.

**Table 4:** Short‐ and long‐term memory performance of the participants and comparisons to reference data

	Range	Mean (SD)	Percentile n (%)			*t*	*P*	*d*
			≤50th	≤30th	≤10th			
15‐Word test direct recall								
*♂*	27‐60	46.47 (9.25)	11 (65%)	5 (29%)	4 (24%)	0.36	.72	0.09
*♀*	30‐63	44.40 (8.77)	18 (72%)	14 (56%)	8 (32%)	3.35	**<.01**	0.66
15‐Word test delayed recall								
*♂*	3‐15	9.35 (3.37)	11 (65%)	8 (47%)	3 (18%)	0.84	.41	0.20
*♀*	3‐15	8.40 (3.14)	18 (72%)	15 (60%)	8 (32%)	3.95	**<.01**	0.79

Bold values represent *p* < .05.

### 3.3 CANTAB tests

On the Motor Screening Task (MOT), all but 2 patients were faster and all but one made fewer errors than reference data. This means that, in general, there was no presence of sensorimotor deficits or lack of comprehension.

**Figure 1:**
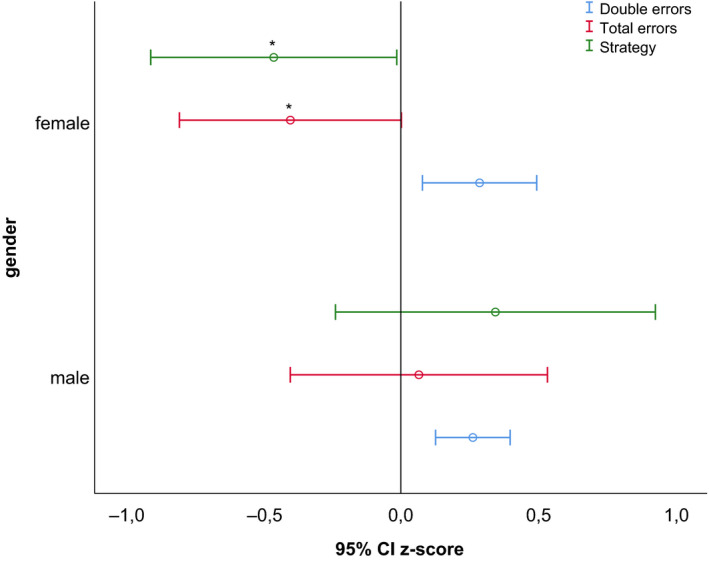
*Z*‐scores of spatial working memory (SWM) subtests: double errors, total errors and strategy in males and females. Data are presented as mean z‐scores and 95% confidence intervals. *P ≤ .05

As shown in Figure 1, female participants scored significantly worse on Spatial Working Memory (SWM) total errors and SWM strategy, with small to medium effect sizes (*d* = 0.41 and 0.43, respectively) when compared to reference data. No differences were found on the SWM between errors subtest. Also, no differences with reference data were found for male participants.

Affective Go/No‐Go (AGN) data are shown in Table 5. Mean affective bias was negative in both men and women, which means that participants had faster reaction times during positive blocks than during negative blocks (a positive bias), which was comparable to reference data. When compared to reference data, men as well as women had significantly slower reaction times on the AGN. Also, women made more errors of commission and omission. This was not found in male participants. Paired *t* tests were conducted to compare negative to positive blocks within the gender subgroups. In the male participant group, more errors of commission were made during positive blocks than during the negative blocks (*t*(15) = 2.83, *P* = .01), but no significant differences were found in latency (*t*(15) = 1.62, *P* = .13) and errors of omission (*t*(15) = 0.20, *P* = .85). Female participants showed slower reaction times during negative blocks than during positive blocks (*t*(24) = 3.20, *P* = <.01), but significant differences were found in errors of commission (*t*(24) = 1.95, *P* = .06) or errors of omission (*t*(24) = 0.31, *P* = .76). Also, paired t tests were conducted to compare set‐shift to nonshift blocks within the gender subgroups. No significant differences were found in latency (*t*(15) = 0.43, *P* = .67), errors of commission (*t*(15) = 0.42, *P* = .68) and errors of omission (*t*(15) = 0.20, *P* = .85) in male participants. These results were also found in women, with no significant differences in latency (*t*(24) = 0.74, *P* = .47), errors of commission (*t*(24) = 1.13, *p* = .27) and errors of omission (*t*(24) = 0.31, *P* = .76).

**Table 5:** Scores on the Affective Go/No‐Go (AGN) and comparisons to reference data

	Hypopituitarism		Reference Mean	*t*	*P*	*d*
	Range	Mean (SD)				
Affective bias						
*♂*	−101.69 to 32.94	−14.73 (36.35)	N/A	N/A	N/A	N/A
*♀*	−104.93 to 45.00	−24.49 (38.42)				
Mean correct latency (msec)						
*♂*	454.25‐740.56	613.77 (69.48)	498.55	6.63	**<.01**	1.66
*♀*	479.45‐800.19	598.00 (77.13)		6.45	**<.01**	1.29
Error of commission						
*♂*	0‐20	7.63 (6.03)	7.14	0.32	.75	0.08
*♀*	1‐30	11.64 (8.08)		2.79	**.01**	0.56
Error of omission						
*♂*	0‐15	6.06 (4.91)	5.14	0.75	.46	0.19
*♀*	0‐31	8.48 (8.13)		2.06	**.05**	0.41

Bold values represent *p* < .05.

## 4 DISCUSSION/CONCLUSION

The aim of the current study was to assess with objective measures a wide spectrum of cognitive functions in patients with different aetiologies of hypopituitarism, at least six months after adequate replacement therapy. We intentionally chose a heterogeneous group of hypopituitarism patients to represent clinical practice and to provide tools for recognition in the outpatient clinic. It was expected that these patients show subnormal scores on neuropsychological tasks. Data of this study show that subjective cognitive functioning is worse in patients treated for hypopituitarism compared to reference data. Interestingly, only female participants seem to notice a higher increase in, more hindrance of, worries about and were more often annoyed by their cognitive dysfunctions. Also, female participants treated for hypopituitarism perform worse on different aspects of memory (working, short‐term and long‐term memory) and executive functioning compared to reference data. These results were not found in male participants. On affective information processing bias, both men and women had slower reaction times and women made more commission and omission errors compared to reference data. This shows that especially in women, focus attention and general competence in inhibiting behavioural responses is worse. Since no differences between set‐shift and nonshift blocks within the participants were found, it seems that participants had no problem in their abilities to suppress and reverse stimulus‐reward associations. Overall, participants reacted faster on positive than negative words, implying a positive bias. A possible explanation for this is that a positive attentional bias could be part of a coping mechanism for dealing with (chronic) illness. Previous research of Caprara et al^31^ found that positivity led to an improvement in quality of life in cancer patients, since positivity was associated with less functional impairment and less negative affect. This suggests that positivity may play an important role in dealing with illness and illness‐related challenges during daily living.

To our knowledge, this is the first time that the CANTAB tests are used in patients with treated hypopituitarism. The CFQ has previously been used once in a group with treated nonfunctioning pituitary adenoma^17^ and 15‐WT in a group with treated acromegaly.^16^ This is the first study that used these cognitive tests in a group with different aetiologies of hypopituitarism.

Yedinak and Fleseriu^18^ assessed patient perceptions of cognitive deficits in 41 patients with (treated) pituitary adenomas: active acromegaly, controlled acromegaly and nonfunctioning pituitary adenoma (NFPA). For each patient group, they found that more than half of the patients reported cognitive dysfunction to some extent. Since the highest levels of self‐reported cognitive dysfunction and severity were found in NFPA, the study indicates that cognitive dysfunction can still be present while hormonal levels are within normal range. A limitation to this cited study is that no objective cognitive variables were measured, so outcomes solely depended on patient’s self‐perception. To our knowledge, our study is the first to assess both subjective and objective cognitive functioning in hypopituitarism. This is of importance since discrepancies between self‐percepted and actual cognitive dysfunction may exist. Remarkable to the results of our study is that patients with hypopituitarism indicate considerable levels of subjective dysfunction, but this dysfunction is not always objectified by neuropsychological tests. While in female participants most outcomes on objective neuropsychological tests were worse, this finding was not found in male participants.

Gender differences in cognitive functioning were found for different aspects of memory (working, short‐term and long‐term memory) and executive functioning. While male participants scored similar as age‐ and gender‐adjusted (and educational‐adjusted) reference data, female participants did score worse. In line with these results, a previous study on memory and well‐being during growth hormone (GH) replacement therapy indicated that spatial working memory was affected by different GH doses in females, but not in males. Authors concluded that their findings indicate that only in female’s cognitive performance relying on frontal lobe functioning benefits from a low‐dose GH treatment.^32^ Combining these results of the present study with those of Van Bunderen et al., we suggest that females are more sensitive to various GH doses because of their subnormal pretreatment cognitive performance. Notably, studies show a higher morbidity and even excess mortality rates in women compared to men within hypopituitarism,^33^, ^34^ implying that female participants may be more affected by hypopituitarism. Another study of Van Bunderen et al^35^ noted an increased mortality rate in treated growth hormone–deficient adults when compared to the general population, but only in women. Olsson and Bengtsson^36^ stated that these differences might be due to the notion that women with hypopituitarism are treated less optimal than men, such as a 15% higher replacement dose of glucocorticoids per kg. Treatment imperfections regarding women have been known for other chronic illnesses. A recent retrospective cohort study of treatment adherence after myocardial infarction in Dutch subjects found that gender differences also existed, and that women did not receive optimal treatment.^37^ Another study of Billimek et al^38^ indicated that for lipid management amongst patients with type 2 diabetes, women had poorer lipid control than men. Another contributing factor that may play a role regarding cognitive decline in women is oestrogen. The neuroprotective actions of oestrogen will decline during loss of this hormone (ie, ageing, postmenopausal or hypopituitarism) and are associated with neuroinflammation, synaptic decline and cognitive impairment.^39^ While results of preventive effects of oestrogen replacement therapy in postmenopausal women on cognitive decline are controversial and contradicting, a study of Girard et al^40^ found that replacement therapy may have a beneficial effect on cognitive control prefrontal mechanisms in this group of patients. More research has to be conducted to clarify of the gender difference in cognition, morbidity and mortality in hypopituitarism.

One of the advantages of this study is that nearly all participants fully completed the test battery; only one participant had missing data on the AGN. Furthermore, most neuropsychological tests were computerized, which limited differences in test taking and made it possible to detect subtle deviations in neuropsychological functioning. The relatively small sample size of this study implies a limitation, since this leads to a reduced statistical power. Unfortunately, no data on premorbid neuropsychological function were available. This makes it impossible to attribute cognitive impairment solely to the hypopituitarism, since it could not be excluded that our group of participants already had some level of cognitive decline before the start of the hypopituitarism. Another limitation is the lack of a matched healthy control group, which made it impossible to make comparisons in cognitive function between hypopituitarism and a population with equal demographic variables. By comparing the results of this study to age‐matched, gender‐matched and educational level matched reference data, we tried to minimalize possible biases. Besides, the number of nonparticipants was large (58%). It could be stated that patients with little concerns about their cognitive functioning are less inclined to participate and thereby leading to bias. On the other hand, testing being too stressful or confronting were amongst mentioned reasons in nonparticipants. Compared to participants, nonparticipants had more congenital forms of hypopituitarism. This could potentially lead to bias, since childhood‐onset hypopituitarism can affect cognitive development^41^ and thereby has a negative effect cognitive functioning. Also, comorbidity was present in 26 (62%) of the participants. Amongst these comorbidities were conditions that can influence cognition, such as type 2 diabetes mellitus and epilepsy.^42^, ^43^ Although patients were stably treated, this could form a potential bias. Besides, five participants used antiepileptics and one participant used an antidepressant, both medications that can influence cognition.^44^, ^45^

Despite the intentional selection of the heterogeneous group of patients with hypopituitarism, this also forms a limitation. Aetiologic groups are represented in small numbers: that is ten participants with nonfunctioning pituitary adenoma, five with acromegaly, five with Cushing’s disease and four prolactinoma. Therefore, the results of this heterogeneous group are difficult to translate to the situation of individual patients. However, we intended to give an overview of what to expect from cognitive functioning in a representative sample of hypopituitarism patients an endocrinologist might see at the outpatient clinic. Also, sensitivity analyses showed that groupwise exclusion of four important subgroups of patients (cranial radiotherapy, congenital forms of hypopituitarism, Cushing’s disease and acromegaly) does not change the subnormal performance of the remaining group of patients (data not shown). Thus, at least individual patients can be expected to have subnormal cognitive functioning. In addition, the present results provide tools for earlier recognition and better understanding and explanation towards the patients. Future research will need to develop appropriate intervention strategies to assist patients suffering from cognitive impairment.

## 5 CONCLUSIONS

Our study is the first to point out that neuropsychological functioning, regarding different aspects of memory and executive functioning, is impaired in a heterogeneous group of patients with treated hypopituitarism. Subjective dysfunction seemed impaired in both men and women but when objectifying the cognitive domains, only women appear to be affected. Hence, it seems that adequate replacement therapy in hypopituitarism does not result in complete recovery of neuropsychological function in females. This study is of importance because it provides essential information for clinical practice about the course of hypopituitarism and the expected long‐term problems on specific subdomains of cognitive functioning, which patients might encounter in general. Current treatment mainly focuses on hormonal disturbances^46^ rather than functional outcomes. Therefore, it is recommended to conduct additional research, which focuses on the design and evaluation of a cognitive remediation therapy. Our study proposes that such an intervention should be directed on compensation of impairments in different aspects of memory and executive functioning. By developing such an intervention, neuropsychological complaints in treated hypopituitarism may decline, which in turn may lead to an improvement of quality of life.

## CONFLICTS OF INTEREST

The authors have no conflicts of interest to declare.

## AUTHORS’ CONTRIBUTIONS

Prof. Dr. M.L. Drent conceived the original study idea. All authors developed the study protocol. T.N.A. Slagboom planned and carried out the experiments, analysed the data and wrote the manuscript. Dr. J.B. Deijen verified the analytical methods. All authors provided critical feedback on the manuscript.

## Data Availability

Research data are not shared.
